# Comparative, Cost and Multi-Criteria Analyses of Traditional Binders in the Composition of Hemp-Based Finishing Products

**DOI:** 10.3390/ma18020452

**Published:** 2025-01-19

**Authors:** Raluca Iștoan, Daniela-Roxana Tămaș-Gavrea, Mihaela Dumitran, Ovidiu Gavriș

**Affiliations:** Department of Civil Engineering and Management, Faculty of Civil Engineering, Technical University of Cluj-Napoca, 28 Memorandumului Str., 400114 Cluj-Napoca, Romania

**Keywords:** traditional binders, mechanical properties, thermal insulation, acoustic absorption, plant waste, natural organic resources

## Abstract

The objective of this paper is to analyze the characteristics of twelve compositions based on hemp shiv and four traditional binders used in the construction industry: cement, plaster, hydrated lime and clay, with the aim of using the resulting materials as final finishing products applicable to the raw area of walls, slabs and other construction elements for walls. Comparative, cost and multi-criteria analyses were carried out on the proposed compositions. The comparative analysis focused on acoustic, thermal, mechanical and fire characteristics, followed by a cost analysis and ending with multi-criteria analysis. In general, cement presented the highest values for mechanical properties, while the other binders demonstrated the most favorable results for acoustic and thermal properties. This paper aims to provide an overview of the traditional binders used in hemp shiv composition and to examine the impact of the physical and mechanical properties of these binders on the final product.

## 1. Introduction

Hemp is a material that is commonly used in the construction industry in two principal forms. The first one: as a closure/partition material, which is known as hempcrete [1,2,3]. This is a composite material comprising hemp shivs (wood fibers) and lime, which serves as a binder. The second one: as an insulating material–a mattress, defined by the textile fibers of the hemp. The use of a binder is optional in this product and depends on the technological process applied [4,5,6].

A review of the scientific literature, including of the studies referenced above [1,2,3,4,5,6], indicates that hemp has significant potential in the construction sector. Some press articles have even characterized this resource as a game changer, the resource of the future, and refer to hemp-based materials as a sustainable building revolution [7,8,9].

However, the properties of hemp composites are influenced by a number of factors, beginning with the type of binder, which plays an essential role in bonding and providing cohesion to the hemp shiv. Additionally, the proportion of binder incorporated, the size of the shiv, the volume of hemp shiv used, the type and the water content, the manufacturing process, the curing regime and the age of the material all contribute to the final properties of the composite [10,11].

In the context of construction, a binder refers to a material which, when mixed with an aggregate (usually sand) and water, behaves similar to an adhesive or bonding agent. This mixture forms a fresh plaster, render, mortar or concrete, which upon setting and curing, can fulfil structural and/or decorative roles within a building [12].

The traditional binders are most commonly defined as clay, gypsum, lime and cement. From a historical view, clay binders date back to 8300 BC and were utilized in the adobe walls of Jericho (Israel) [13]; gypsum-based mortars and plasters have had a long history as masonry coverings and supports for mural paintings [13]; lime has been identified in the floors and pavements of ruins excavated in Çatalhöyük, dated between 10,000 and 5000 BC [13]; and cement, which was found in Ancient Rome, dated from the late 2nd century to the mid-1st century B.C, in the concrete structures in ports along the central Italian coast [14]. Binding agents such as hydraulic lime and Roman cement were common in the Byzantine, Medieval, Renaissance and Baroque periods after the fall of the Roman Empire. Portland cement was later introduced in the early 19th century by Aspdin [13].

The use of traditional binders, which are widely available around the world, and hemp, which is easy to grow without special cultivation conditions, could facilitate the technological process of obtaining products with similar properties on a global scale, thereby eliminating the need to use specific binders. This approach would improve the sustainability of the construction sector, by reducing the CO_2_ emissions associated with transporting raw material. Aligned with this approach, this paper presents data based on comparative, cost and multi-criteria analyses.

The main focus was to investigate how the different ratios of hemp with selected binders affect mechanical, thermal and acoustic properties, providing insights into the potential applications of hemp in construction.

## 2. Materials and Methods

### 2.1. Materials

Hemp used in the samples was purchased from Hempflax Romania. The increase in hemp production in Europe from 2015 to 2022, with an 84.3% growth [15], reflects a significant interest in adopting hemp as a sustainable crop, while in Romania, the area dedicated to hemp cultivation reached 3400 hectares in 2018, positioning the country as the fifth in Europe for hemp cultivation [16]. This growing interest in hemp served as the starting point for the research, with the objective of determining the impact of hemp in association with available market binders to obtain finishing products for the construction industry.

The comparative analysis was based on 4 binders, of which 3 commercial ones were used: white cement from Italcementi (Bergamo, Italy), gypsum from Rigips (Bucureşti, Romania), hydrated lime from Carmeuse (Brașov, Romania) and clay—a non-commercial one that was collected near the Făget area, dried for 2 days at a constant temperature of 105 °C and ground to a powder [14,15].

Additional information about the binders is analyzed in Table 1 [17].

The selection of the four binders was based on the following aspects:

- Availability: The selected binders were readily available on a global scale, ensuring a consistent supply for a variety of construction applications.
- Affordability: These binders were cost-effective, making them accessible for a wide range of construction projects.
- Architectural Applications: As finishing products, the final compositions would not require additional coatings or chemicals, offering a natural, smooth finish for surfaces. However, it was also important to maintain the porosity of the binders for optimal sound absorption properties. The selection of white cement was based on its aesthetic and acoustic properties.
- Binder properties: Firstly, clay and lime were selected to enhance thermal performance, thereby improving the energy efficiency of buildings. Secondly, gypsum was chosen for its rapid installation, which contributes to accelerated construction timelines. Finally, cement was proposed for its durability, ensuring that the final product can endure over time and in various environmental conditions. Although the use of cement can be controversial due to its high carbon footprint, the proposal of this binder in the current study is to know how it works as an individual part.

### 2.2. Sample Mix Designs

For each binder, three mix designs were analyzed, in which the hemp/binder ratio gradually increased in volume from 1:1 to 3:1. The binder/water ratio was kept at a constant level of 0.5, even when the amount of hemp in the composition increased.

All samples were maintained under identical conditions: a humidity level of 55% and an indoor temperature of 23 °C during 28 days for hemp–cement and hemp–gypsum composites, and up to 90 days for hemp–lime and hemp–clay composites. Table 2 presents the ratio of the hemp-based composite materials and the references where they were presented in the previous studies. As documented in the mentioned table [18,19,20,21], these data have previously been examined for each binder individually for all or some of the mentioned parameters: acoustic (A), thermal (T), mechanical (M) and fire (F). However, the novelty of this work was to perform a comparative analysis of all four binders and to complement this with a cost analysis and multi-criteria analysis to determine the best performing composition.

**Figure 1:**
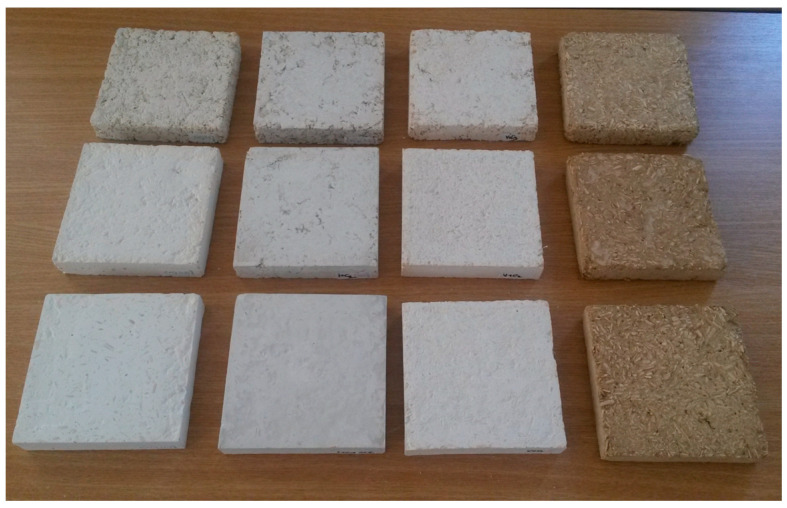
Samples of the twelve recipes.

The 12 samples are presented in Figure 1. Starting from the left-hand side, the compositions are as follows: cement and hemp; gypsum (plaster) and hemp; hydrated lime and hemp; and clay (earth) and hemp. The ratio of the binder–hemp is 1:1 at the bottom, 1:3 in the middle and 1:3 at the top.

### 2.3. Methods

#### 2.3.1. Comparative Analysis

The comparative analysis focused on the physical–mechanical properties defined by the sound absorption coefficient, density, thermal conductivity, compressive strength, flexural strength and fire behavior. The acoustic and thermal properties of a material influence the comfort of the living space, while the mechanical properties determine their durability, workability and installation process. It is also important to consider fire resistance in a living space, as it offers the potential for greater safety.

The parameters, standards, equipment and samples used are presented in Table 3, Table 4 and Table 5.

#### 2.3.2. Cost Analysis

The cost of materials used in construction is influenced by the cost structure, determined by the technological process involved. It includes the following:

- Technological costs: represent the basic components such as the purchase and use of direct materials (e.g., hemp fiber, binder, additives).
- Labor costs: reflect wages, social security contributions and other taxes related to workers, and varying according to the complexity of the process and the level of specialization.
- Machinery and equipment costs: include depreciation, maintenance and the energy consumption of equipment used in production (e.g., mixing, application).
- Additional costs: taxes, environmental certification, performance testing and other quality assurance costs.
- Overheads: administrative costs, logistical coordination, marketing and other activities necessary for production and market launch.

Pricing for sustainable materials, such as those made from hemp, is complex, involving high demands for quality, sustainability and compliance with environmental standards, which is why the price calculation for the current composite materials were based only on technological cost. The cost was established after considering the 2023 binding offer provided by the hemp producer and the market price of the binders.

#### 2.3.3. Multi-Criteria Analysis

Materials are designed to serve a variety of functions and are developed to accommodate multiple parameters. The multi-criteria methods are designed to evaluate and select the most suitable alternative(s) from a set of options, taking into account a number of criteria for assessment. This approach facilitates the identification of the most optimal material selection for building performance, environmental impact and occupant well-being [26,27,28]. For the current study the multi-criteria method of global percentage weighting was applied [29].

The applied criteria were based on data collected from current research:

- Physico-mechanical characteristics: acoustic (on standardized frequency bands 250, 500, 1000, 2000, 4000 Hz), thermal (density and thermal conductivity), mechanical (compression and flexural strength) and fire.
- Cost.

Each criterion was ranged based on the best performing/best price, such as the highest sound absorption, lowest density and thermal conductivity, highest compressive and flexural strength, highest fire resistance and lowest price.

The evaluation criteria are applied in turn to each product under consideration. A scoring system, such as a 1 to 10 scale, is then employed to assign a rating to each product in each category, reflecting the extent of its success. Thereafter, the global weight for each product is calculated by multiplying the scores by the assigned weights for each criterion; this total score represents the weighted value for each product. In the final analysis, the product that has accumulated the highest score is regarded as the optimal choice, taking into account all of the factors.

In the current analysis, the criteria were assigned equal weighting, in accordance with the distribution set out in Table 6. According to this analysis, the objective was to determine the optimal composition from the twelve composite materials, taking into account all the parameters studied.

Starting from the weighting criteria presented in Table 4, the research aimed to analyze the new materials from two perspectives: firstly, on the basis of their acoustic and thermal properties and, secondly, on the basis of the applicability of the binders.

In the first perspective, more weight was given to acoustic and thermal properties compared to other attributes, without neglecting the mechanical properties.

The second approach focused on examining the binders used in various contexts, emphasizing the versatility of hemp-based materials.

These materials are not limited to a single application but are suitable for a wide range of scenarios. From the construction of green housing prioritizing sustainability to the restoration of historic buildings requiring materials with specific thermal and acoustic properties, hemp-based composites offer innovative solutions.

Their adaptability further extends to industrial applications, such as prefabricated modular units, and to urban housing, where their natural properties improve indoor comfort. Multi-criteria analysis was used to comprehensively assess these aspects.

## 3. Results and Discussion

In order to facilitate an accurate interpretation of the graphs presented in this chapter, a list of material names is provided in Table 7 for reference.

### 3.1. Comparative Analysis Results

#### 3.1.1. Acoustic Properties

**Figure 2:**
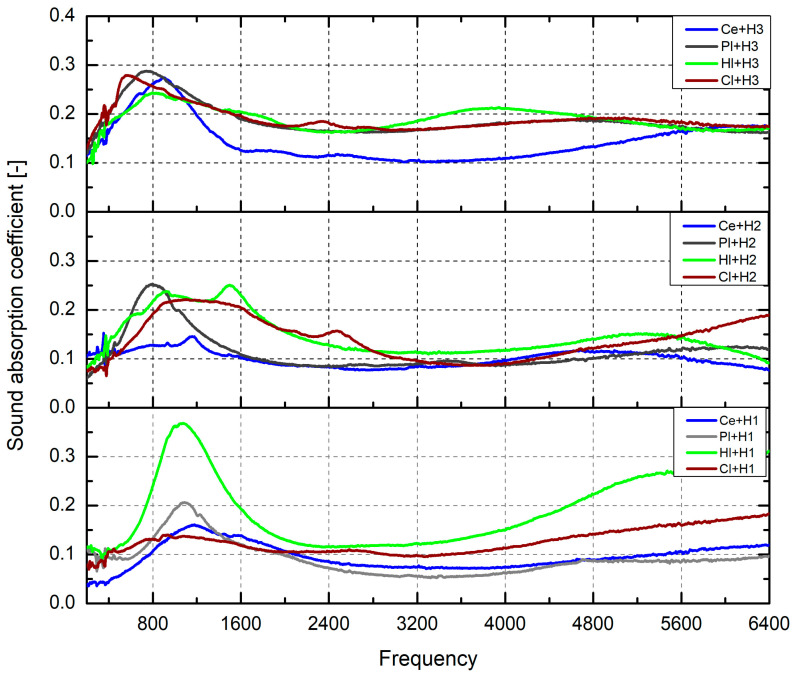
Sound absorption coefficient.

**Figure 3:**
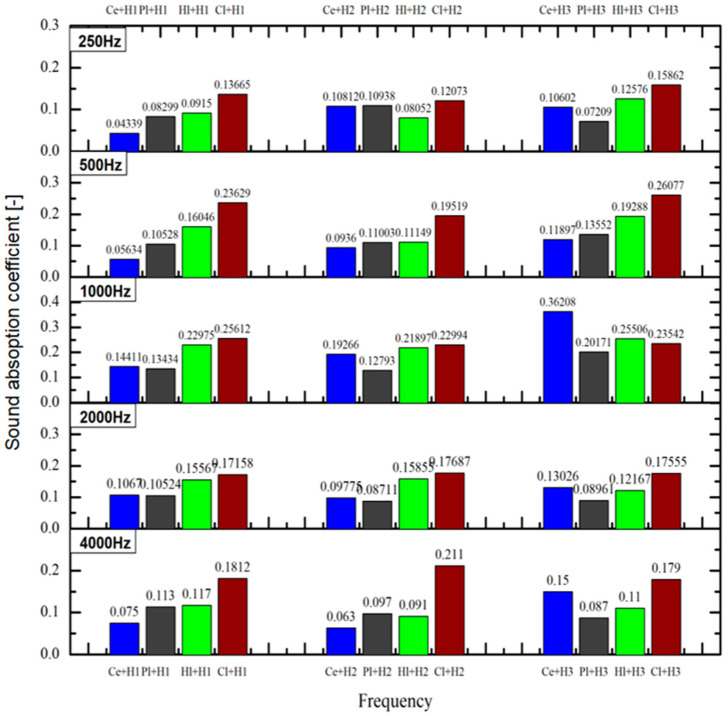
Sound absorption coefficient on standardized frequency bands.

The values for the sound absorption coefficient are presented in Figure 2 and Figure 3. The first figure presents the values obtained for each Category within the frequency range of 0–6400 Hz, while the second figure presents the data for the sound absorption coefficient across the standardized frequency bands of 250 Hz, 500 Hz, 1000 Hz, 2000 Hz and 4000 Hz.

The results presented in Figure 2 indicated that the highest peak for Category I (bottom of the figure) was approximately 0.3, which corresponded to an absorption rate of approximately 30% at a frequency of 800 Hz. This value was associated with the composition in which clay was present. In comparison to the other binders, the peak was observed to follow a descending order, with hydrated lime, then cement and plaster.

The highest values of sound absorption for Category I were observed within the frequency range of 500–2400 Hz. In the case of Category II (middle figure), the highest peak was approximately 0.25, which was defined by the same composition as clay. However, the tendency for this composition was to exhibit a similar absorption rate across the entire frequency range. The decreasing performance of the binders were similar in Category I: clay, hydrated lime, cement and plaster.

In Category III (top of the figure), an higher peak was observed for the composition with cement at a frequency of 1000 Hz, which then rapidly decreased until reaching a frequency of 2400 Hz. Thereafter, an increase was noted, starting around 4000 Hz. For this Category, the highest value was around 0.4, and it is important to note that for the other binders, the values of the sound absorption peak were mostly identical, around 0.28.

The data provided in Figure 3 on the standard frequency bands indicated an increasing trend in Category I for the sound absorption coefficient values at 250 Hz, 500 Hz and 4000 Hz, beginning with the composition of cement, then plaster, hydrated lime and clay. However, a variation was observed at the 1000 Hz and 2000 Hz bands, where cement had the lowest value.

At the ratio of 2:1 hemp to binder for Category II of the composite materials, all of the binders showed similar values of sound absorption; however, just for the bands at 500 Hz and 4000 Hz, the clay composition values were more notable than the other materials.

For Category III, the most optimal behavior was defined by the cement–hemp composition, having the highest value at 1000 Hz, while the other standardized frequency bands showed the highest values for the clay–hemp composition.

It would be necessary to inquire why a hemp–cement composition indicated an improvement of acoustic characteristics in Category III, given that the ratio of hemp to cement was 3:1. A possible reason for this situation is the increased porous structure of the composition, which leads to an internal structure in which sound waves are scattered and lose energy, thus increasing the absorption.

The understanding of the sound absorption coefficient values from the current study required a comparison with those found in the scientific literature [30,31,32,33,34] and are presented in Table 8. This evaluation should consider the impact of different binder compositions and densities on the acoustic performance of the materials at varying frequencies. The objective is to facilitate the determination of the performance of each material in absorbing sound and to support the selection of appropriate binders for specific acoustic applications.

From the scientific literature, the composites based on hemp–cement had high absorption coefficient values in the 1000 Hz segment according to the study [34], particularly between 0.65–0.95, and with increasing frequency; towards high frequencies, the absorption coefficient decreased and fell between 0.35–0.5. In cement and lime-based composites [33], a maximum alpha at 700 HZ was obtained with an absorption value of 0.9. Compared to the present study, the aforementioned values were better performing; in the current study, the maximum absorption capacity of the materials was not higher than 40% or more than 0.4.

The study [30] indicated that the acoustic absorption properties of hemp–clay and hemp–lime were dependent on density, with the following reported ranges: 180–195 kg/m^3^, 339–340 kg/m^3^ and 461–470 kg/m^3^. An increase in density affected both materials in a similar manner; the absorption peak shifted to lower frequencies with a lower amplitude. The acoustic behavior of hemp–clay and hemp–lime mixtures was comparable. Both the hemp–clay and hemp–lime samples were tested in three ways, defined as light, medium and heavy compaction. The samples with clay showed the maximum absorption: α_maxcl_ = 1 at 1000 Hz for light, α_maxcm_ = 0.75 at 600 Hz for medium and α_maxch_ = 0.4 at 2000 Hz. Based on the current study, the values of the hemp–clay composite arrived at a maximum of 0.3 on low frequency (700–800 Hz) in all three cases.

In the case of hemp–lime composites, the scientific studies presented a variety of potential matrix options for the binder, with a recommended absorption range of 0.2–0.6 depending on the frequency [28]. These values were comparable to those of the current study, where the absorption range was between 0.2 and 0.3, and also to the results of [30], where the maximum for heavy compaction was approximately 0.2 and the absorption profile was similar to that observed in the current study. When the compaction was medium or light, the absorption increased to approximately 0.82 at 600 Hz and 0.98 at 1000 Hz.

A review of the scientific literature revealed no studies investigating the use of hemp–gypsum composites. However, the current study presented values for the absorption range between 0.1 and 0.2, with optimal performance observed at lower frequencies.

The hemp–clay composites exhibited higher absorption coefficients across all plots in the current study at mid-to-high frequencies, indicating that this configuration is more effective in absorbing sound within those ranges.

#### 3.1.2. Thermal Properties

**Figure 4:**
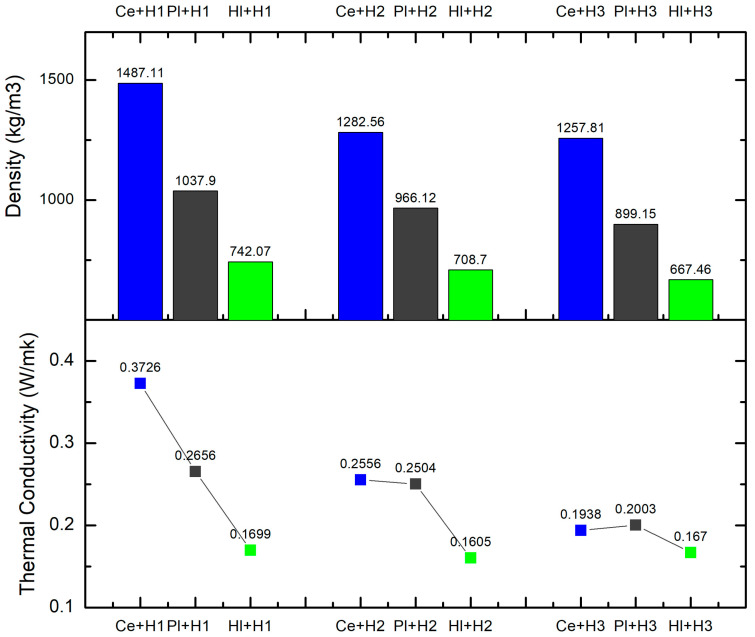
Density and thermal conductivity.

For the current research, the thermal properties of a composite material were defined in terms of density and thermal conductivity. In accordance with Figure 4, it was observed that no values were available for the clay composition, as the determination was not possible at 90 days, when the samples exhibited weak, fragile behavior.

The results showed a decrease in density in all three categories, namely cement, plaster and hydrated lime. The lowest density values were observed in the hydrated lime compositions, which may be attributed to the reduced quantity of powder incorporated into the matrix of the novel composite materials. The density of the cement-based composition was approximately 1200–1600 kg/m^3^, whereas the values for the hydrated lime-based composition were approximately half those of the cement-based composition.

In contrast with the density, the thermal conductivity presented close values for the plaster and hydrated lime in all the three categories.

It is important to note the situation of the hemp–cement composition, which decreased the density values by almost 50% when increasing the hemp ration compared to the binder. In this situation, where the density remained relatively constant but thermal conductivity decreased significantly, further investigation was interrogated. An explanation of the raw materials and how they were expressed in the matrix composition of the new composite materials can be provided. The amounts expressed in kilograms did not show noticeable variation in the mixture of composite materials when expressed in volumes. However, an improvement in thermal conductivity was observed for the cement samples. By increasing the hemp volume, the composite materials increased their thermal conductivity performance, becoming better than gypsum in the third part, where the hemp–cement sample had a 3:1 ratio. This trend could be explained in the case of cement, by the fact that increasing the hemp volume may contribute to reducing the pore size between the hemp shivs and subsequently, the sample became more compact.

The density of hemp-based materials can influence their thermal and mechanical properties. Accordingly, the values for hemp-based composite materials and different binders have been collected from previous studies published in the scientific literature and are summarized in Table 9 [34,35,36,37,38,39,40,41,42].

In the scientific literature, hemp and lime-based composites showed a range of densities and thermal conductivity values that varied based on the specific recipes used. For most studies, the density of these composites generally fell between 374 and 1750 kg/m^3^, with thermal conductivity values ranging from 0.08 to 0.135 W/mK.

When focusing on lime and hemp composites specifically, Williams et al. obtained densities between 374 and 432 kg/m^3^, with corresponding thermal conductivities between 0.08 and 0.13 W/mK [41]. Similarly, Šadzevičius et al. recorded a density of 482 kg/m^3^ and a thermal conductivity of 0.114 W/mK [39], while Horszczaruk et al. reported a density of 655 kg/m^3^ with a conductivity of 0.12 W/mK [40]. Subanesh et al. found slightly higher values, with a density of 780 kg/m^3^ and a conductivity of 0.135 W/mK [42]. In the current study, the composite reached a density of approximately 700 kg/m^3^, with thermal conductivities ranging from 0.1605 to 0.1699 W/mK. Although these values were slightly higher than previous findings, they still fall within a comparable range, supporting the suitability of hemp–lime composites in applications requiring moderate thermal insulation.

For hemp–clay composites, the current study was unable to derive any meaningful data as these composites were too fragile to be tested. In addition, there was a lack of research in the literature that provided information on this type of material.

In contrast, for hemp–gypsum composites with densities between 900–1050 kg/m^3^ and thermal conductivities between 0.2003 and 0.2656 W/mK, this study agreed with the results of study [36], which reported densities around 500–1000 kg/m^3^ and thermal conductivities between 0.14 and 0.3 W/mK. However, study [35] showed even higher performance with densities of 366–392 kg/m^3^ and thermal conductivities of 0.098–0.102 W/mK.

For hemp–cement-based materials, the present study recorded density values at least 10% higher than any previously documented, in the range of 1300–1500 kg/m^3^. Thermal conductivity values were comparable to previous studies, particularly those approaching 0.2 W/mK.

#### 3.1.3. Mechanical Properties

The mechanical characteristics, both flexural (*f*ctm) and compressive (*f*cm) strengthswere plotted not on the basis of the three categories of materials reported for the binder group (as initially was presented for the acoustic and thermal properties), but by each binder individually for the three situations of 1:1, 1:2 and 1:3. The explanation for this lies in the values obtained, and the different intervals between them, making graphical representation difficult.

##### Compressive Strength

**Figure 5:**
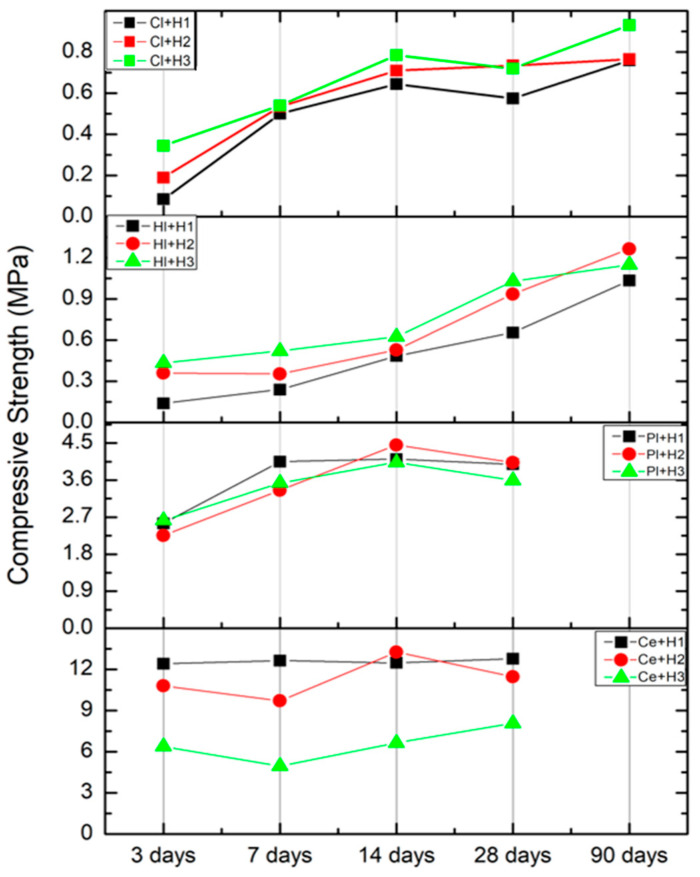
Compressive strength.

Figure 5 illustrates the values obtained at 3, 7, 14 and 28 days for all the binders, with the addition of 90-day data for clay and lime.

As shown by the data, the best performing composition of hemp–cement was Ce + H1, which approached 12.5 MPa, while those of gypsum plaster Pl + H2 achieved 4 MPa, with both values presented at 28 days. The best value for the lime compositions was due to Hl + H2, which presented a value of 1.2 MPa, and for clay Cl + H3 at around 0.9 MPa at 90 days.

In the case of the first binder, cement (Figure 5-bottom), the transition from one category to the other was characterized by significant variation. A reduction in the volume of hemp resulted in a notable improvement in the compression performance. In contrast, the categories for plaster exhibited a relatively consistent pattern across all testing days. However, the behaviors of lime and clay were markedly different. In these cases, the composition with the highest volume of hemp demonstrated the most favorable performance across a range of testing days.

Hemp-based materials utilized as plaster finishes are classified as non-load-bearing elements and are typically characterized by the lowest values in compressive strength. In fact, even hempcrete, which is defined as hemp concrete, is utilized as insulation materials without exhibiting any significant load-bearing characteristics.

A comparative analysis of the data from the current study with the scientific literature [11,35,36,38,39,40,41,42,43,44] is presented in Table 10, which outlines the compressive strength of various hemp composite materials.

The majority of studies identified in the literature focused on the hemp–lime composite, which is typically defined as hempcrete. The data for compressive strength in this Category exhibited a range of 0.15 to 3.85 MPa, varying according to the percentage of lime in combination with other binders. Looking only at studies where the materials were 100% lime, the values of compressive strength reported included 0.693 MPa [39], 2.64 MPa [42], 0.15–0.45 MPa [41], and 2.56 MPa [40]. When these results were viewed in terms of material density, there was a clear trend that higher density correlated with higher compressive strength. In comparison, data from recent research on lime-based materials with densities between 600–800 kg/m^3^ showed compressive strengths at 90 days ranging from 0.9 to 1.2 MPa—approximately 50% lower than those reported in existing studies. A possible reason for this discrepancy may be the storage conditions: unlike other studies, these samples were stored in a dry laboratory environment with no additional humidity control, which may have influenced the results.

A review of the literature revealed a lack of uniformity and correlation in the reported values for hemp and cement-based composite materials. This makes it challenging to establish a relationship between the reported values and the density of the final products. For example, in the study conducted by Sahin [11], the values of compression strength ranged from 0.28 to 1.24 MPa for densities between 312 and 928 kg/m^3^. In the study conducted by Horszczaruk et al., the values for a cement-based recipe with a density of 988 kg/m^3^ reached a strength value of 9.56 MPa. Furthermore, the study conducted by Subanesh et al. reported relatively low strength values of 2.97 MPa observed at a density of 1228 kg/m^3^. In the case of the current study, the cement-based material exhibited densities between 1200 and 1500 kg/m^3^, with compressive strength values ranging from 7 to 12 MPa at 28 days. Therefore, these values were approximately three to four times higher than those reported in previous studies for composites with similar densities.

In compositions using clay earth as a binder with hemp, compression strengths have been documented as between 0.39 and 0.48 MPa, with corresponding densities ranging from 370 to 510 kg/m^3^, as reported by Clay Brahim Mazhoud et al. [44]. These findings aligned with the data obtained for the three categories of samples in the current research, where compressive strengths ranged between 0.1 and 0.9 MPa.

In the category of samples composed of hemp and gypsum plaster, previous studies reported compressive strengths ranging from 0.28 to 0.55 MPa, with an associated density of approximately 380 kg/m^3^. In the present study, the densities were approximately 50% higher than those reported in previous studies, and the compressive strengths were significantly greater, ranging from 3.5 to 4.5 MPa—approximately eight to nine times higher than previously documented values.

##### Flexural Strength

**Figure 6:**
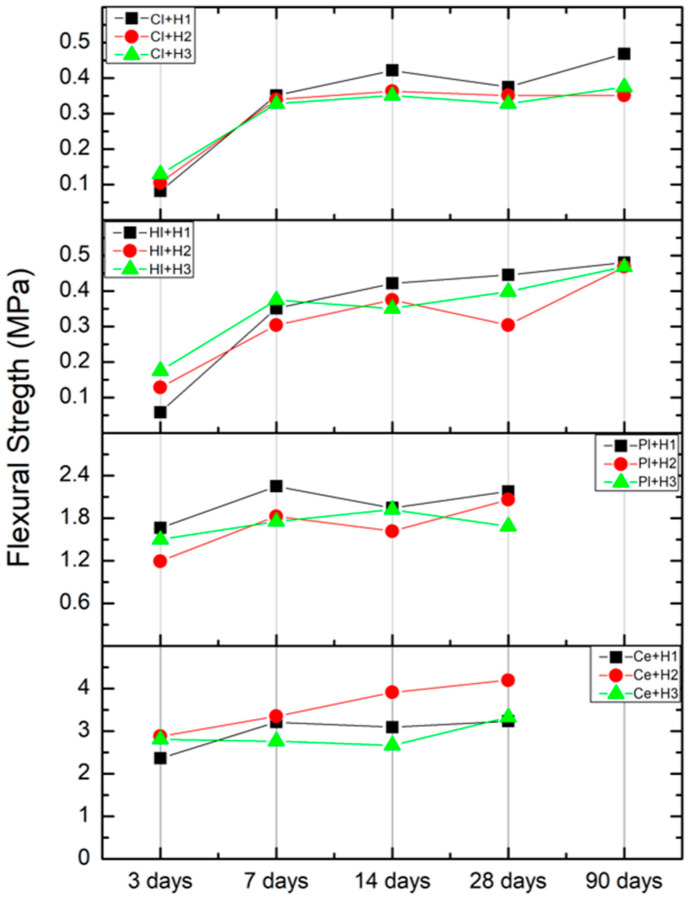
Flexural strength.

Figure 6 presents the data collected at the same time interval as that used in the comparative strength diagrams.

The values for cement exhibited a range of 2–4 MPa. The composition Ce + H2 demonstrated optimal performance at 28 days. The results for the hemp–plaster materials ranged from 1.2 to 2.4 MPa. The composition Pl + H1 showed the optimum performance compared to the three compositions during all the test periods. With regards to the values of lime and clay composite materials, where the values ranged between 0.1 to 0.5 MPa, it was observed that the best performing materials were the ones with the lowest volume of hemp (Hl + H1 and Cl + H1).

Flexural strength is a fundamental property of materials utilized in applications where bending or tensile forces may be encountered, such as in the fabrication of panels and boards. The data obtained from the scientific literature on hemp-based materials are presented in Table 11 [41,42,43,44,45]. The composite materials, which consisted of hemp wood fiber (hemp shiv) and various types of binders at varying percentages, demonstrated flexural strength values ranging from 0.02 to 1.5 MPa.

The present research findings suggested that hemp–cement composite materials exhibited higher values, approximately 50% higher than those observed in previous studies [42,45].

The hemp–lime materials from the ongoing study demonstrated a range of values between 0.05 and 0.5 MPa, which were comparable to the findings of Williams et al. [38], where the values ranged from 0.1 to 0.3 MPa. However, flexural strength exhibited the lowest values in comparison to the study conducted by Subanesh et al. [42].

The investigation carried out by Mazhoud et al. regarding hemp–clay composites revealed values between 0.02 and 0.026 MPa, which were the lowest recorded compared to the present study where the highest values reached 0.5 MPa [44].

Despite an exhaustive search, no data regarding hemp–gypsum composites were found. However, based on the findings of the current study, the range values of flexural strength were determined to be between 1.2 and 2.2 MPa.

### 3.2. Fire Properties

**Figure 7:**
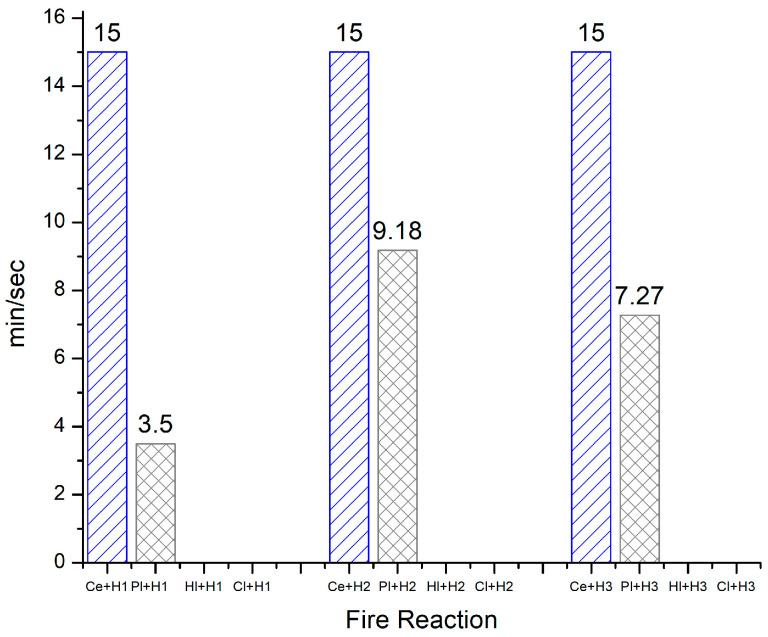
Fire reaction.

Fire properties were examined in accordance with the method for cohesion of the core at bending during an exposure temperature of 950 °C for 15 min. Therefore, the results obtained were only for cement and plaster composites; the lime and clay composites failed before the determination started (Figure 7). For the two remaining binders, cement was constant throughout the three recipes, completing the test at 15 min without collapsing, while for plaster, the highest performance was found for the Pl + H_2_ composition, as well as for the Pl + H_3_ composition, which were both resistant for 50% of the test time.

In the production process, the impact of binders on global warming was found to be six times higher for cement and five times higher for lime in comparison to gypsum [46].

Therefore, with regard to the finished products based on hemp and binders, the study by [47] presented building blocks derived from hemp and lime, formulated at a ratio of 40% lime to 60% hemp with a density of 650 kg/m^3^, as being highly resistant to combustion. These findings suggest that the organic composition of these products does not significantly contribute to fire behavior.

With regard to the fire behavior of hemp–clay-earth- and hemp–gypsum-based concretes, the study [46] indicated that their flammability is primarily influenced by the density of the final products, with the type of binder playing a less significant role. Accordingly, the research indicated that low-density concretes exhibited a higher ignition rate, although the flame was not sustained for more than 60 s. The flame diminished after less than one minute, did not markedly accelerate the decomposition rate over an extended period, and the residual fraction was not affected by ignition. The lightest compositions can be considered to be minimally affected by thermal processes, as heat transfer from the surface to the mass was negligible. The production of smoke was always insignificant for these concretes [46].

### 3.3. Cost Estimation

**Figure 8:**
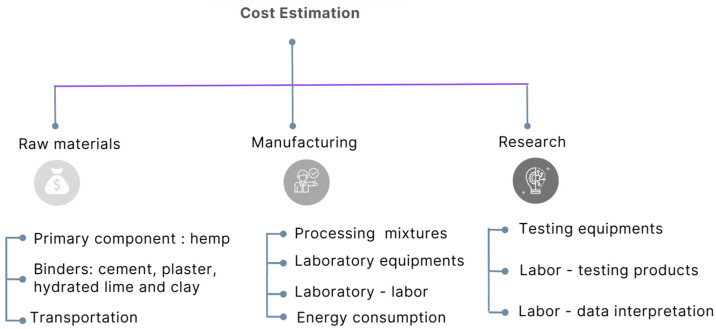
Cost estimation product diagram.

The diagram below, Figure 8, provides an illustration of the cost structure that forms the basis for the financial outcome of the composite products analyzed in this paper.

The cost structure was based on three principal elements: raw materials, manufacturing and research. It was not the intention to define the research and manufacturing aspects at this stage; however, they must be taken into account when formulating the final price list. The present study quantified only the direct costs of the raw materials (hemp and binders) procured directly from various suppliers.

**Figure 9:**
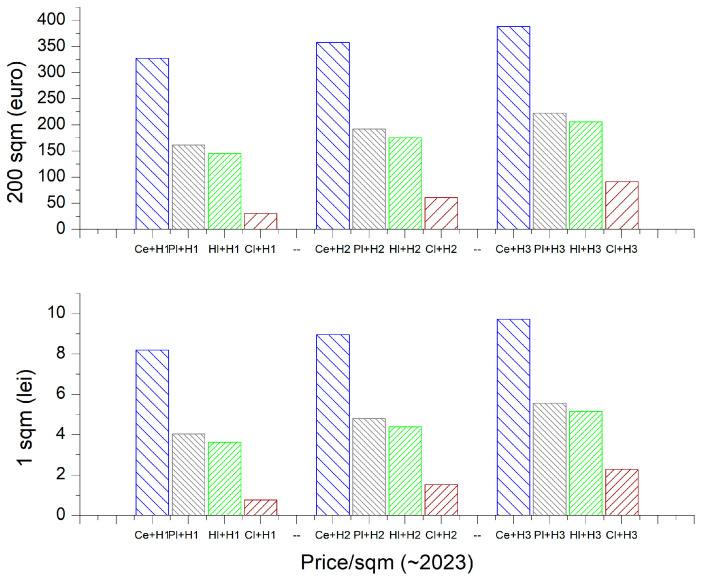
Cost estimation.

In Figure 9, the prices of the composite materials were expressed for one square meter (in local currency) and 200 square meters (in euros). The 200 m^2^ option was basically the amount of mortar for a ground floor house with dimensions of 11 m × 9.6 m and a floor height of 2.65 m. The division of the house included two rooms, a living room, a technical and dressing room, a kitchen and a bathroom. On the cost chart, it could be seen that the mixtures with cement are the most expensive, due to the fact that white cement was used, which usually has a higher price than the normal Portland cement. The price of hydrated lime and gypsum composites was 50% lower than hemp–cement composites. For the clay composites, the price was based only on the primary resource, as the clay was sourced directly from the land.

However, the final diagram of the costs can be rearranged when considering the transport of the raw materials, which was not included in the price, and the modality of obtaining the powder of clay, which implied using the oven, drying the clay, sorting it and all of the processes described in the article [18].

A review of the scientific literature revealed a notable absence of data concerning the price/cost composition of hemp-based building materials. The only information identified in this regard was the cost of hemp insulation, which was determined to be approximately 6.1 euros/m^2^ [48].

### 3.4. Multi-Criteria Analysis

In consideration of the data presented in Section 2.3, it can be concluded that an equal distribution of the weight throughout the parameters studied indicated that the optimal composition of the 12 proposed combinations was Cl + H3. Table 12 outlines the optimal compositions, where the sum of the values exceeds 1, starting with Cl + H3; Cl + H2; Ce + H3; Cl + H1; and Ce + H1. It was observed that even the clay-based compositions were all in the first positions; however, the compositions could not be overlooked, as they also demonstrated a notable score and should therefore be considered.

Based on the outcomes of the previous phase, it was proposed to continue with the values and to determine the optimal composition when increasing the weight of the parameters related to the acoustic and thermal properties of these materials. As evidenced in Table 13, the initial variant (V1) demonstrated a 50% enhancement in acoustic properties, while maintaining thermal and fire properties at 20% and reducing those related to mechanical properties and price to 5%. This was justified by the classification of these materials as non-structural elements, where price is not a determining factor in the classification of conforming performances of the final products. In the following variant (V2), the weight assigned to thermal performance was modified, increasing to 50%.

**Figure 10:**
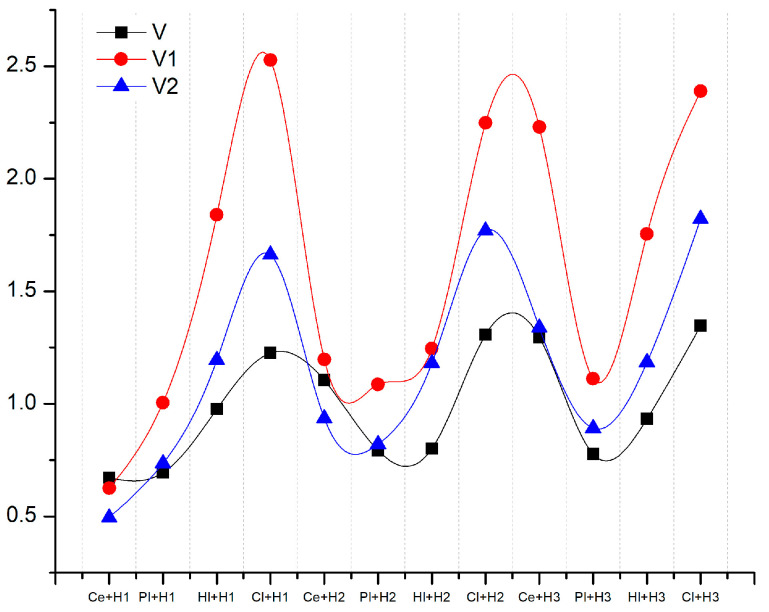
Variation 1.

Figure 10 illustrates the data generated based on the variants proposed in Table 13.

It can be observed that when the weight of the acoustic and thermal parameters was increased, the Cl + H1 composition became optimal in both variants. However, in the case of favorable acoustic properties (V1), the clay- and cement-based materials became the most optimal, while on the thermal side (V2), the values for clay- and lime-based compositions increased. Another notable distinction among the three scenarios was that in variant V, the second cement category tended to be the most favorable in comparison to the other compositions. As the weighting increased in V1 and V2, the composition was outperformed on the acoustic side (V1) by gypsum and on the thermal side (V2) by lime.

Furthermore, the impact of focusing solely on the acoustic properties and assigning greater weight to this segment was examined. Table 14 presents a comparison of the proposed variants with the basic variant. Three options were proposed, with the increase made by 60, 70 and 80%, respectively. In variant A1, the remaining parameters were assigned equal weights of 10%. In variant A2, the weight decreased to 5% for cost and fire. In variant A3, the weights for fire and cost were eliminated entirely. The complete elimination of the weighting for fire was implemented because only cement and plaster were available for testing, and the cost was not intended to be a significant factor at this stage.

**Figure 11:**
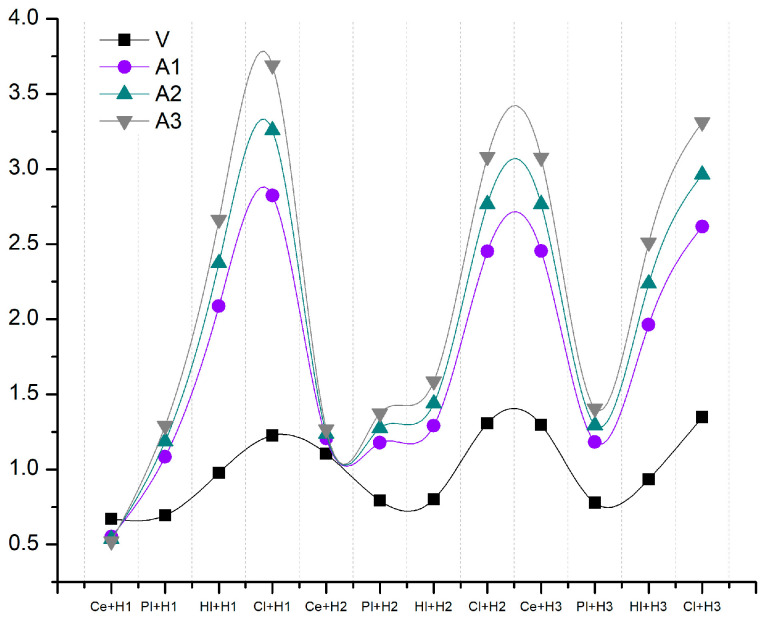
Increasing acoustic weight.

Figure 11 illustrates the outcomes of the proposed variants in Table 14, specifically regarding the augmentation of the acoustic properties’ weight. In the case of the three variants (Cl1-Cl3), it was evident that the peaks were more pronounced, the values were considerably higher, and the trends remained consistent across the same composite materials. The only points of intersection or points in close proximity between the basic variant and the acoustic variants were in categories 1 and 2 in the cement composite. A gradual increase in binder-based composite materials is observed, with the most optimal being clay-based in Categories I and II and cement-based in Category III.

Table 15 repeats the structure from Table 14, but this time the focus is on the thermal properties of the hemp base materials.

**Figure 12:**
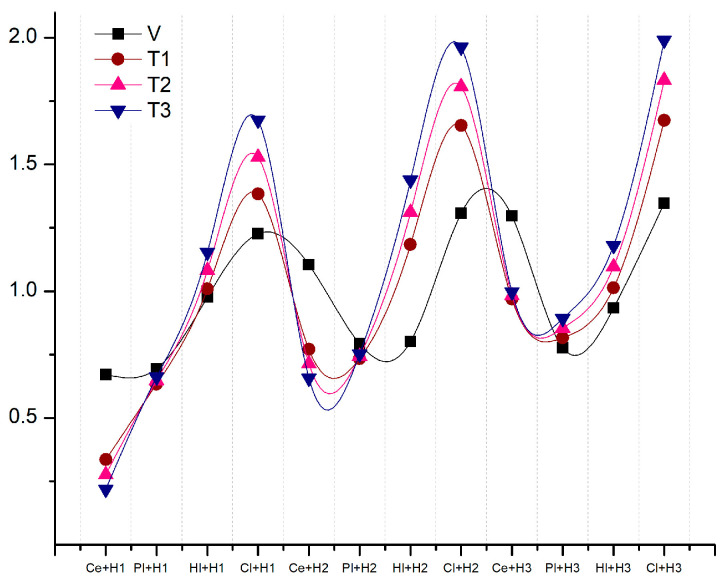
Increasing thermal weight.

Figure 12, based on Table 15, illustrates the enhancement of thermal properties in the composite materials and their evaluation in comparison to the fundamental variant, where all parameters were assigned equal weight. The data revealed that, across all three categories, clay-based materials were the most optimal, followed closely by those with lime. The Ce + H2 composition of the standard variant exhibited a decline in ranking as the weight assigned to thermal properties increased, ultimately being surpassed by lime-based compositions. It can thus be argued that clay- and lime-based compositions represented optimal performance.

A second approach to the application of the multi-criteria method based on global weights can be made on the premise of the space in which the hemp-based composites will be applied as finished end products. As illustrated in Table 16, the cost was eliminated, underscoring the paramount importance of acoustic and thermal properties, while not compromising the role of mechanical and fire resistance properties. The weight percentages allocated to acoustic and thermal properties were 40%, while those assigned to mechanical and fire resistance were 10%.

For the ecohouses option, the selected finishing products were based on clay and hydrated lime–hemp composites, whereas for the traditional houses or urban spaces, the most common finishing binders were cement, gypsum and hydrated lime.

**Figure 13:**
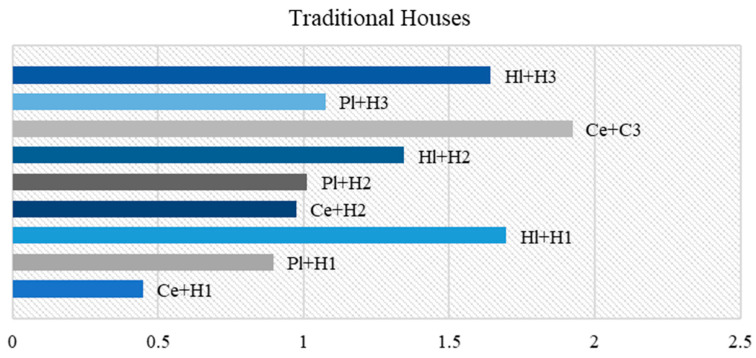
Best performing finishing products for traditional houses.

Figure 13 illustrates that the most effective finishing material for traditional spaces were those based on cement with a proportion of hemp increased by up to three times the standard ratio. It is notable that the composite based on hydrated lime and hemp with a 1:1 ratio was the second most performant material from this analysis, exhibiting a performance that was very close to that of the composite with a 3:1 ratio of hemp to lime. Among the other results, it could be observed that the sample with gypsum Pl + H1 could be classified as exhibiting a similar performance to that of Pl + H2 and Ce + H2.

**Figure 14:**
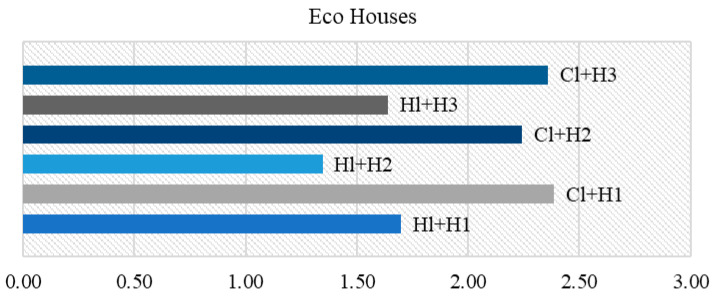
Best performing finishing products for eco houses.

Figure 14 illustrates the performance of the most effective material among the binders proposed for the construction of ecological houses, such as clay and lime. It can be observed that the most efficient composition was Cl + H3, however, in general, all clay compositions demonstrated superior efficacy compared to lime. Upon interpreting each category of composite, it became evident that the same pattern was observed for both binders. Category I demonstrated the most favorable performance, followed by Category III and then Category II.

### 3.5. Results Synthesis

The comparative analysis of hemp-based materials showed that products made with lime or clay were primarily suited for coatings and plasters, while those containing cement or plaster were better for tiles due to their higher strength. Hemp fibers reinforce these materials, increasing their strength and reducing cracking. Additionally, hemp’s porous structure enhanced the thermal and acoustic performance of the materials. As a renewable and biodegradable resource, hemp is ideal for creating eco-friendly construction products.

As such, the multi-criteria evaluation method facilitated the selection of the most appropriate binder based on their performance, highlighting the trade-offs between mechanical, thermal and acoustic performance.

- Lime–hemp composites exhibited thermal and acoustic performance, which proposed them to be used in two principal applications: as finishes in historical renovations, due to their breathability and aesthetic compatibility with traditional materials, and in new house construction, where their low environmental impact and high thermal efficiency stand out as key advantages. Lime-based hemp compositions exhibited a compressive strength around 0.9–1.2 MPa with a flexibility 0.5 MPa and tended to be less prone to cracking over time. Lime’s ability to absorb CO_2_ during curing, combined with hemp’s CO_2_ absorption during growth, makes these compositions highly attractive for low-carbon construction. Due to the porous nature of both lime and hemp, these materials, based on their compaction mode, could offer significant thermal and acoustic advantages, with results from the current study showing ρ reaching 0.16 W/mk and α = 0.28. Although lime requires a longer setting time than other hydraulic binders, the final aesthetic appearance is superior. Cost related aspects: in general, the cost of lime–hemp materials is higher than cement–hemp ones, but in the current paper the cost of lime-based composites was the lowest among the commercial binders, because the cement–hemp-based composites were developed with white cement.
- Clay–hemp composites are proposed to be used in green homes, as both hemp and clay are considered sustainable products that meet global development requirements. Upon reaching the end of their life cycle, these materials do not present any adverse effects to humans or the environment and can be integrated into a circular economy without any issues. The clay-based composite had the lowest compressive strength of the binders at 0.8 MPa, but this is usually sufficient for non-load bearing interior finishes. In terms of thermal and acoustic performance, the binder is known to be highly breathable, and with its natural porosity it performs better compared to the other binders, shown for this study with an α_max_ = 0.3. With the best environmental impact, being considered the most environmentally friendly, easy to find and with low energy consumption, clay is easier to process and has a quick application, requiring constant moisture, which is more suitable for warmer areas. Widely, people choose clay because of its earthy properties that connect the home more with nature, but also because the cost is relatively inexpensive, especially in areas where it can be sourced locally, as was the case in this study.
- Cement–hemp composites could be utilized in industrial applications due to their increased mechanical properties. With a higher compressive strength of 8–12 MPa higher than the other binders analyzed, cement-based composites are shown to be more suitable for applications requiring higher structural support. In terms of thermal and acoustic properties, when the volume of hemp was increased by three times more than the binder, the conductivity reached 0.1938 W/mK and the αmax achieved values higher than 0.4, making these materials more suitable for insulation than the other composites. As a binder, cement is easier to apply with no workability restrictions but can be more prone to cracking over time when temperatures are fluctuating. Even with good performance, this material is still being discussed because of its significant negative environmental impact, but in terms of cost, it is cheaper and more widely available than lime. In the present study, the cost was higher than the other binders because white cement was used.
- Gypsum–hemp composites are well-suited for urban finishes, providing breathable spaces with a contemporary appearance. As a binder, gypsum plaster presented moderate values for compressive strength 3.5–4 MPa and around 2 MPa for flexural strength. It is not recommended to be used for wet or high-traffic areas, which is why the composite based on gypsum will perform better in indoor space, where the temperature is controlled, having a conductivity around 0.200–0.250 W/mK and a maximum absorption around 0.25. The workability of these materials depends on the way the material is applied, as it could be an advantage working in a precast way, better than applied directly onto construction elements, as it has a quick setting time. Interiors where plaster has been used are defined by a clean and smooth finish, giving the space a modern touch. Looking at the binder in terms of its environmental impact, it could be included somewhere between cement and lime, but ultimately it is more difficult to recycle than clay binder. In terms of cost, this binder is widely available, and it is chosen mainly for indoor applications.

## 4. Conclusions

This study provides a detailed analysis of composite materials made from hemp and traditional binders, exploring their potential performance in the construction sector. In addition to their physico-mechanical characteristics, the proposed composite materials were evaluated from a cost perspective under laboratory conditions. Furthermore, this study applied a multi-criteria decision-making method to identify the optimal mixture, thereby ensuring that both performance and economic feasibility were taken into account in the selection process. Based on the three aspects, it outlined the following aspects that the hemp-based composites exhibited variability in terms of their strength, thermal and acoustic performance. Cement–hemp demonstrated structural excellence but carried a substantial environmental impact. Conversely, lime–hemp and clay–hemp are eco-friendly and ideal for finishes and insulation; and gypsum–hemp is suitable for indoor aesthetics. Clay–hemp was the most sustainable and cost-effective option, while lime-hemp offered low-carbon benefits.

Future research will concentrate on the development of materials with improved strength and insulation, while maintaining a low environmental impact and optimized hemp–binder ratios to improve mechanical, thermal and acoustic performance across diverse climates and applications.

## Figures and Tables

**Table 1:** Binders’ characteristics.

Properties	White Cement	Hydrated Lime	Plaster	Clay
Real density [kg/m^3^]	2630	2000	2500	2500
Bulk density [kg/m^3^]	1024	520	810	1130
Compactness [%]	38.7	26	32.4	45.2
Porosity [%]	61.3	74	67.6	54.8

**Table 2:** Ratio of hemp–binder composite materials.

Sample Mix Designs	Ratio Binder—Hemp in Volume			References	Parameters
Cement–Hemp	1:1	1:2	1:3	[21]	A, T, M, F
Hydrated Lime–Hemp				[19]	A, T
Gypsum plaster–Hemp				[20]	A, T, M, F
Clay earth–Hemp				[18]	A, T, M

**Table 3:** Evaluated parameters.

Properties	Parameter	Standard
Acoustic Properties	Sound absorption coefficient	SR EN ISO 10534-2:2002 [22]
Thermal properties	Density	SR EN 998-1 [23]
	Thermal conductivity	Norm C107/3 [24]
Mechanical properties	Compressive strength	SR EN 998-1 [23]
	Flexural strength	SR EN 998-1 [23]
Fire properties	Core cohesion at bending	SR EN 520+A1:2010 [25]

**Table 4:** Devices.

Parameter	Sound Absorption Coefficient	Density	Thermal Conductivity	Compressive Strength	Flexural Strength	Core Cohesion at Bending
Device Name	Kundt tube	Electronic balance	FOX 200 Heat Flow Meter	Hydraulic press	Fruhling–Michalis device	Fire resistance testing device
Site Testing	Transilvania University of Brasov	Technical University of Cluj-Napoca	Technical University of Cluj-Napoca	Technical University of Cluj-Napoca	Technical University of Cluj-Napoca	Rigips Factory of Turda
Photo	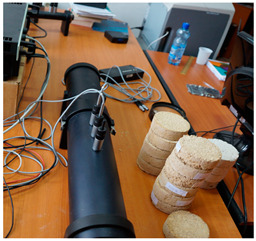	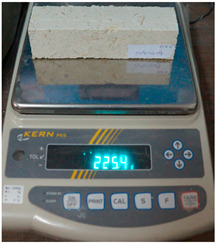	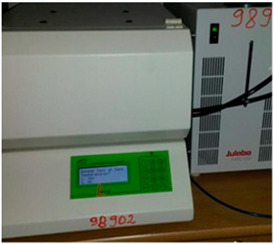	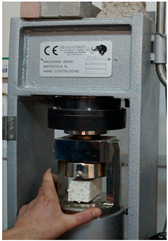	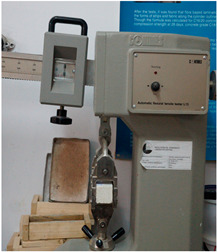	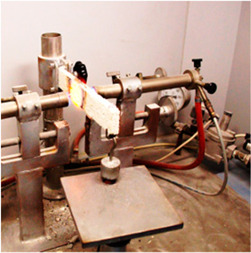

**Table 5:** Samples.

Parameter	UM	Sample Shape	Sample Size	Testing Days
Sound absorption coefficient	Hz	Cylinder	Ø100 [mm] and Ø28 [mm] h_sample_ = 3 mm	After 28 days
Density	Kg/m^3^	Cuboid	150 × 150 × 30 [mm]	After 28 days
Thermal conductivity	W/m^2^ K	Cuboid	150 × 150 × 30 [mm]	After 28 days
Compressive strength	MPa	Rectangular prism	40 × 40 × 160 [mm]	At 3, 7, 14, 28/90 days
Flexural strength	MPa	Rectangular prism	40 × 40 × 160 [mm]	At 3, 7, 14, 28/90 days
Core cohesion at bending	15 min at 950 °C	Lamellar	45 × 300 × 12 [mm]	After 28 days
Sample photos	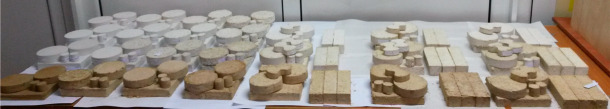			

**Table 6:** Criteria weight (%).

Main Criteria	Sub Criteria	(%)
Physico-mechanical characteristics	Acoustic properties	20
	Thermal properties	20
	Mechanical properties	20
	Fire properties	20
Cost		20
**TOTAL**	**100%**	

**Table 7:** Abbreviation Names of Composite Materials.

Binder	Organic Resource	Category/Ratio in Volume (Binder: Hemp)		
		I/(1:1)	II/(1:2)	III/(1:3)
Cement (Ce)	Hemp Shiv (H)	Ce + H1	CE + H2	CE + H3
Plaster (Pl)		Pl + H1	Pl + H2	Pl + H3
Hydrated lime (Hl)		Hl + H1	Hl + H2	Hl + H3
Clay (Cl)		Cl + H1	Cl + H2	Cl + H3

**Table 8:** Data of sound absorption coefficients from the scientific literature.

Binders	α_500Hz_	α_1000Hz_	α_2000Hz_	α_max_	Reference
Clay	0.3–0.7	0.35–1	0.4–0.8		[30]
Lime	0.2–0.8	0.2–0.9	0.25–0.6		[30]
Hydrated lime + pozzolans	0.42–0.52	0.37–0.45	0.41–0.53		[31]
Hydraulic lime	0.32–0.45	0.24–0.37	0.26–0.39		[31]
Without binder	0.4	0.85	0.57	α_900Hz_ = 0.95	[32]
White Portland cement + Lime	0.05–0.65	0.08–0.68	-	α_700Hz_ = 0.9	[33]
70% CL90s, 20% NHL3.5, 10% CEM I	0.32	0.24	0.26		[31]
100% commercial binder	0.45	0.7	0.39		[31]
70% CL90s, 30% GGBS	0.49	0.42	0.44		[31]
80% CL90s, 20% metakaolin	0.46	0.39	0.44		[31]
70% CL90s, 30% GGBS, 0.5% methyl cellulose	0.52	0.45	0.53		[31]
80% CL90s, 20% GGBS, 0.5% methyl cellulose	0.42	0.37	0.41		[31]
Prompt Natural Cement	0.25–0.5	0.65–0.95	0.35–0.5		[34]

**Table 9:** Data of thermal properties from the scientific literature.

Binder	ρ [kg/m^3^]	l [W/mK]	Reference
Gypsum plaster	366–392	0.098–0.102	[35]
Gypsum plaster	500–1000	0.14–0.3	[36]
Lime–cement	443.02–561.43	0.0965–0.1119	[37]
Magnesium oxysulfate (MOS) cement	411 ± 7	0.113 ± 0.001	[38]
Lime (100%)	482	0.114	[39]
Lime (90%)—Cement II 42.5 (10%)	497	0.108	[39]
Cement HC3	1026 + 11	0.21	[40]
Lime HC1	655 + 22	0.12	[40]
Cement HC2 60% + Lime 40%	988 + 17	0.25	[40]
Prompt Natural Cement	340–415	0.1–0.2	[34]
Lime—Tradical	374–432	0.08–0.13	[41]
Lime—100%	780	0.135	[42]
Lime—100% (Na_2_SO4 = 2% of lime = 22 g)	882	0.129	[42]
Lime—50%, Gypsum—50%	920	0.127	[42]
Lime—90%, Metakaolin—10%	1454	0.119	[42]
Lime—80%, Metakaolin—20%	1750	0.113	[42]
Cement—100%	1228	0.137	[42]

**Table 10:** Data of compression strength from the scientific literature.

Binder	Density [kg/m^3^]	Compressive Strength	Reference
Gypsum plaster	366–392	0.28–0.55 MPa	[35]
Gypsum plaster	500–1000	0.16–0.25 KN	[36]
Cement HC3	988	9.56 Pa	[40]
Lime HC1	655	2.56 MPa	[40]
Cement HC2 60% + Lime 40%	1022	8.74 MPa	[40]
Magnesium oxysulfate (MOS) cement	411	0.7 MPa	[38]
Portland cement	312–928	0.28–1.24 MPa	[11]
Lime (100%)	482	0.693 MPa	[39]
Lime (90%)-Cement II 42.5 (10%)	497	0.698 MPa	[39]
Cement-Hydrated lime	-	1.1 MPa	[43]
Lime—Tradical	374–432	0.15–0.45 MPa	[41]
Clay	370–510	0.39–0.48	[44]
Lime—100%	780	2.64	[42]
Lime—100% (Na_2_SO4 = 2% of lime = 22 g)	882	2.68	[42]
Lime—50%, Gypsum—50%	920	2.70	[42]
Lime—90%, Metakaolin—10%	1454	3.21	[42]
Lime—80%, Metakaolin—20%	1750	3.85	[42]
Cement—100%	1228	2.97	[42]

**Table 11:** Data of flexural strength from the scientific literature.

Binder	Rf [ MPa]	Ref
Cement	1	[45]
Cement-Hydrated lime	0.12	[43]
Lime—Tradical	0.1–0.3	[41]
Clay	0.02–0.026	[44]
Lime—100%	1.137	[42]
Lime—100% (Na_2_SO_4_ = 2% of lime = 22 g)	1.141	[42]
Lime—50%, Gypsum—50%	1.150	[42]
Lime—90%, Metakaolin—10%	1.254	[42]
Lime—80%, Metakaolin—20%	1.373	[42]
Cement—100%	1.216	[42]

**Table 12:** Calculation of percentage weighting.

Composites Mixtures	Physico-Mechanical Characteristics										Cost	Sum
	Acoustic					Thermal		Mechanical		Fire		
	Standardized Frequency Bands					Density	Thermal Conductivity	Compressive Strength	Flexural Strength			
	250 Hz	500 Hz	1000 Hz	2000 Hz	4000 Hz	(Kg/m^3^)	(W/mK)	(MPa)	(MPa)	Minutes	lei/m^2^	
	MAXIM					MINIM	MINIM	MAXIM	MAXIM	MAXIM	MINIM	
Ce + H1	0.00	0.00	0.02	0.04	0.03	0.00	0.00	0.20	0.15	0.20	0.03	0.67
Pl + H1	0.13	0.05	0.00	0.04	0.07	0.06	0.05	0.06	0.06	0.05	0.13	0.69
Hl + H1	0.15	0.10	0.15	0.16	0.08	0.10	0.11	0.00	0.00	0.00	0.13	0.98
Cl + H1	0.17	0.18	0.20	0.18	0.16	0.20	0.11	0.00	0.00	0.00	0.03	1.23
Ce + H2	0.12	0.03	0.09	0.02	0.00	0.03	0.06	0.18	0.18	0.20	0.20	1.11
Pl + H2	0.12	0.05	0.09	0.00	0.05	0.07	0.06	0.06	0.06	0.12	0.11	0.79
Hl + H2	0.07	0.05	0.04	0.16	0.04	0.20	0.11	0.01	0.01	0.00	0.12	0.80
Cl + H2	0.13	0.14	0.05	0.20	0.20	0.20	0.20	0.00	0.00	0.00	0.18	1.31
Ce + H3	0.12	0.06	0.07	0.35	0.12	0.03	0.10	0.12	0.12	0.20	0.00	1.30
Pl + H3	0.05	0.08	0.04	0.11	0.04	0.08	0.09	0.05	0.05	0.10	0.09	0.78
Hl + H3	0.15	0.13	0.05	0.20	0.07	0.11	0.11	0.01	0.01	0.00	0.10	0.93
Cl + H3	0.20	0.20	0.05	0.17	0.16	0.20	0.20	0.00	0.00	0.00	0.17	1.35
Weight	0.2					0.2		0.2		0.2	0.2	1.00

**Table 13:** Weight criteria for acoustic and thermal properties.

Sub Criteria	(%)	Variation 1	
	V	V1	V2
Acoustic properties	20	**50**	20
Thermal properties	20	20	**50**
Mechanical properties	20	5	5
Fire properties	20	20	20
Cost	20	5	5
**Total**	**100%**	**100%**	**100%**

**Table 14:** Increasing weight criteria for acoustic properties.

Sub Criteria	(%)	Variation 2—Acoustic		
	V	A1	A2	A3
Acoustic properties	20	**60**	**70**	**80**
Thermal properties	20	10	10	10
Mechanical properties	20	10	10	10
Fire properties	20	10	5	0
Cost	20	10	5	0
**Total**	**100%**	**100%**	**100%**	**100%**

**Table 15:** Increasing weight criteria for thermal properties.

Sub Criteria	(%)	Variation 3—Thermal		
	V	T1	T2	T3
Acoustic properties	20	10	10	10
Thermal properties	20	**60**	**70**	**80**
Mechanical properties	20	10	10	10
Fire properties	20	10	5	0
Cost	20	10	5	0
**Total**	**100%**	**100%**	**100%**	**100%**

**Table 16:** Weight criteria for different space requirements.

	Percentage	Eco Houses	Traditional Houses
Acoustic properties	40	Clay–Hemp Hydrated lime–Hemp	Cement–Hemp Gypsum–Hemp Hydrated Lime–Hemp
Thermal properties	40		
Mechanical properties	10		
Fire properties	10		
**Total**	**100%**		

## Data Availability

The original contributions presented in this study are included in the article. Further inquiries can be directed to the corresponding authors.
